# Stroke associated with *Mycoplasma hominis* infection: a case report

**DOI:** 10.1186/s13256-021-02903-5

**Published:** 2021-06-15

**Authors:** Anthoula C. Tsolaki, Galaktion Konstantinidis, Stavroula Koukou, Fotini Michali, Despina Georgiadou, Thomas Tegos, Nikolaos D. Michalis

**Affiliations:** 1 Neurology Department, 1st General Hospital of Thessaloniki “Agios Pavlos”, Thessaloniki, Greece; 2 3rd Neurology Department, Aristotle University of Thessaloniki, Thessaloniki, Greece; 3 Radiology Department, 1st General Hospital of Thessaloniki “Agios Pavlos”, Thessaloniki, Greece; 4 Present Address: 1st Neurology Department, Aristotle University of Thessaloniki, Thessaloniki, Greece

**Keywords:** Cerebral infarction, *Mycoplasma hominis*, Thrombosis, Vasculitis stroke

## Abstract

**Background:**

Mycoplasmas are the smallest prokaryotic microorganisms in nature. Many cases of stroke post-Mycoplasma pneumoniae infection have been reported, particularly in the pediatric population. However, *Mycoplasma hominis* infection has not previously been associated with stroke.

**Case presentation:**

We report the case of a 36-year-old Greek woman who presented with an extensive stroke with an unspecified cause. She had a concurrent genital infection with *Mycoplasma hominis* for an unknown duration.

**Conclusion:**

An association may exist between stroke and the immune response to *Mycoplasma hominis* infection.

## Introduction

Stroke is a leading cause of preventable death and adult disability. Infections have been associated with stroke but are not considered directly causal. Their importance has been highlighted in patients with stroke of undetermined etiology and in specific patient populations, such as young patients without traditional risk factors and immunocompromised patients [1].

The overall reported prevalence of acute infections ranges from 18 to 40% in the month preceding acute ischemic stroke onset, and from 10 to 40% in the week preceding stroke. This association has been attributed to multiple immunohematologic alterations leading to plaque rupture and a procoagulant state [2].

None of the studies performed to date have demonstrated a causal role of infection in ischemic stroke. However, our case report suggests that an infection may trigger and temporarily increase the risk of ischemic stroke.

## Case presentation

A 36-year-old Greek woman presented with acute onset dizziness and vomiting. She did not have a history of alcohol/substance abuse or sexually transmitted diseases. The patient was unmarried. According to her family, she was single, without a known medical history requiring medication at the time of the incident. She was a heavy smoker (two packs/day). No known obstetrics and gynecology history was provided by her sister, other than polycystic ovarian syndrome, which had started in the patient’s adolescence. The patient was working as a caregiver for an older couple and living with them 24/7 when the stroke occurred.

Initially, she consulted a local physician in a regional hospital. She was treated with diphenhydramine intramuscular (IM) and was discharged when her symptoms improved. She was also prescribed amoxicillin-clavulanic acid antibiotic tablets acid to treat two suprapubic skin ulcers.

Two days later, the patient became lethargic and was admitted to the hospital emergency department for further investigation. In her first examination, the patient had a fluctuating Glasgow Coma Scale (GCS) score in the range of 7–8/15 and miotic pupils. She opened her eyes only with painful stimulation, had no verbal response and rarely made incomprehensible sounds, and withdrew from painful stimuli. She could not cooperate, but she was able to move all her limbs against gravity, and presented horizontal nystagmus and esophoria of the right eye. Her plantar reflexes were normal, and her tendon reflexes were symmetric on both sides. Her blood pressure at admission was 175/100 mmHg, her pulse rate was 95 bpm, and she had no fever (T = 36.8 °C).

Brain computed tomography (CT) revealed a hypodense lesion on the left cerebellum hemisphere exerting pressure on the 4th ventricle.

The neurosurgical assessment indicated a need for close monitoring, an magnetic resonance imaging (MRI) and re-evaluation in 12 hours if her clinical status remained stable. The patient was treated with bolus mannitol to decrease cerebral edema, and she regained consciousness (GCS 15/15). Her neurological examination after she regained consciousness revealed pupils of the same size with a normal photokinetic response. She had horizontal nystagmus to the left, left facial palsy and diplopia to the left gaze, whereas she could not converge and gaze downward. No clear paresis of the upper or lower limbs was detected, and no sensation abnormality was observed, although the patient’s cooperation was limited. She was unable to sit or stand because of extreme dizziness and nausea. She did not have a fever, and she had an sPO_2_ of 95%, heart rate of 99 bpm, respiratory rate of 15 breaths/minutes, normal arterial blood gas values and fingerstick glucose of 208 mg/dl.

**Fig. 1:**
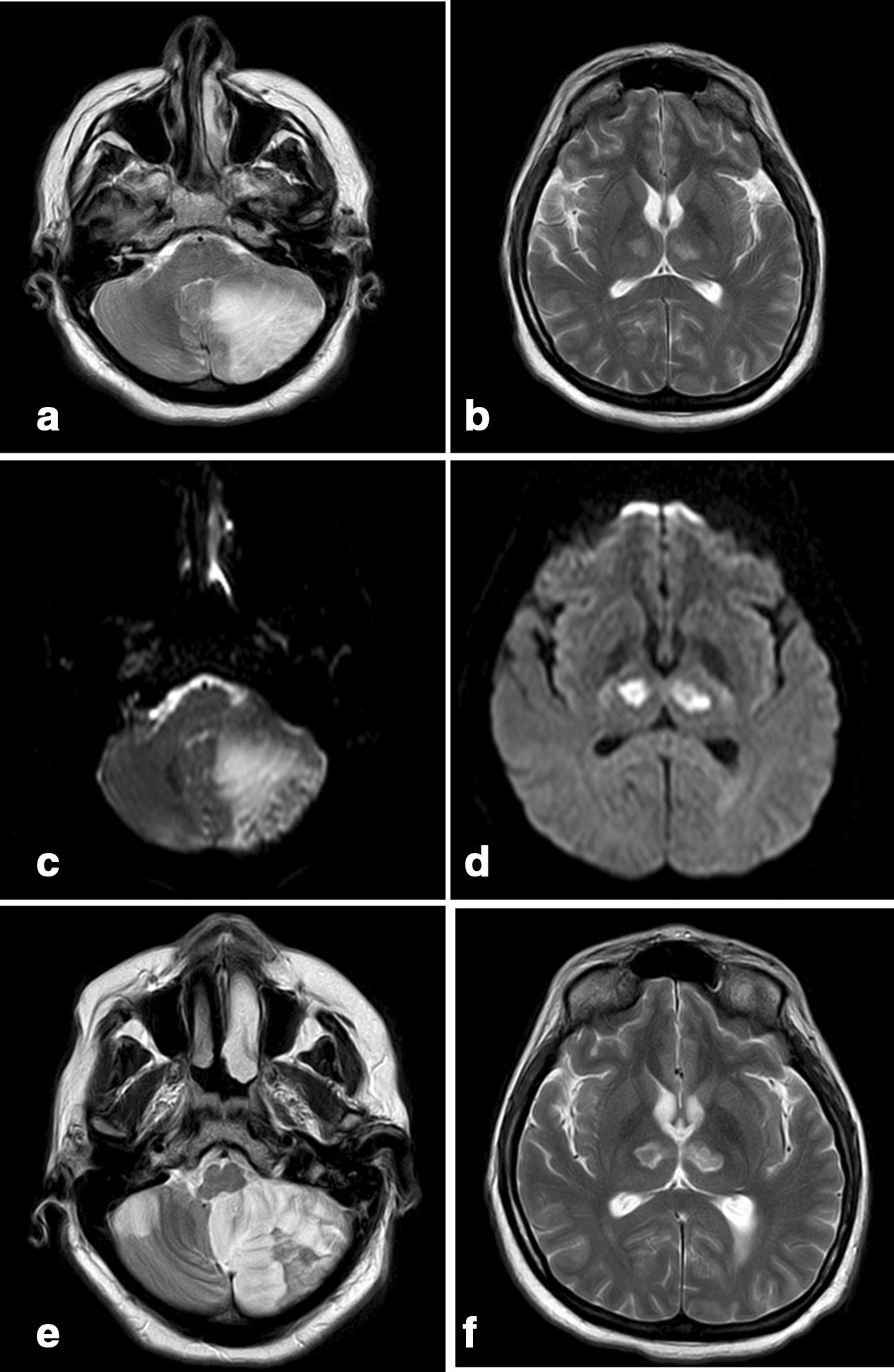
T2-weighted MRI showing a large infarct in the left cerebellar hemisphere (**a**) and rounded areas with high signal alteration in the medial thalami, corresponding to a bilateral paramedian thalamic infarct (**b**). Diffusion-weighted MRI demonstrating the left cerebellar (**c**) and bilateral thalamic infarcts (**d**). Second magnetic resonance imaging (MRI) 8 days later (**e**, **f**), T2-weighted MRI showing a new cerebellar infarct in the cerebellum’s right hemisphere (**e**)

A brain MRI demonstrated an acute ischemic stroke with limited hemorrhagic components in the left cerebellum hemisphere and the left part of the vermis, and pressure on the 4th ventricle. Symmetric thalamic infarcts were also observed. On the 7th-day post-admission, mild deterioration with projectile vomiting was reported, and a new MRI was performed (Fig. 1). A new smaller acute ischemic stroke was found in the right cerebellum hemisphere. An MRI of the intra and extra-cranial vessels performed several days later did not reveal any vascular abnormalities.

Her thoracic X-ray on admission showed pleural effusion, and after a thoracic CT scan, a thoracentesis was performed.

The pleural effusion was small and bilateral, and it met the criteria for exudate (pleural fluid protein: serum protein > 0.5, pleural fluid lactate dehydrogenase [LDH]: serum LDH > 0.6 and pleural LDH > 2/3 the upper normal limit for serum LDH). The pleural effusion pH was 7.6, and the glucose was 90 mg/dl (similar to the plasma value at that time). Few cells were observed, 60% of which were of polynuclear type. The pleural effusion culture was positive for Pseudomonas aeruginosa. However, biopathology and internal medicine consultants considered this to be a false positive result attributable to a contamination due to the sample collection and culture procedure, because the patient had no fever, and no symptoms or signs of pulmonary infection or cancer, on the basis of imaging and laboratory investigation.

Although the patient had no pulmonary infection signs, the possible presence of pseudomonas was not ignored, and the antibiotic therapy was modified to ciprofloxacin according to the antibiogram results. A cardiologic investigation with 24-hour heart rate monitoring, and transthoracic and transesophageal echo was performed to rule out stroke cardioembolic origin, and the results were normal. The aortic arch was normal.

Laboratory tests for autoimmune inflammatory diseases (quantitative evaluation of immunoglobulins, c-Antineutrophil Cytoplasmic Antibodies [ANCA], p-ANCA, Antinuclear Antibodies [ANA], anti-double stranded DNA [anti-ds-DNA], anti-Extractable Nuclear Antigen Antibodies [ENA], anti-Ro, anti-La, anti-Sm, anti-RNP [Ribonucleoprotein], C3, C4, anti-Beta 2 Glycoprotein (β2GPI) and anti-cardiolipin (IgG-IgM) were also negative. The coagulation of antithrombin-3, protein-C, free PS, APC-resistance and anticoagulant test screen results were also negative. The homocysteine was within normal limits (Table 1).

**Table 1:** Patient’s laboratory test results in admission and at discharge

Test normal values	In admission	Discharge	Test	In admission	Discharge
WBC 3.8–10.5 K/μL	11.14	7.35	Glucose 74–106 mg/dl	222	98
RBC 4.20–6.30 M/μL	4.93	5.04	Urea 16.6–48.5 mg/dl	15	26
HGB 14.0–18.0 g/dL	12.8	13.2	Creatinine 0.7–1.2 mg/dl	0.76	0.84
HCT 40.0–52.0 %	40.0	41.8	K^+^ 3.5–5.1 mmol/l	4.5	4.6
MCV 80.0–99.0 fL	81.1	82.9	Na^+^ 136–145 mmol/l	137	137
MCH 27.0–32.0 pg	26.0	26.2	Ca ^2+^ 8.6–10.2 mg/dl	9	10.0
MCHC 32.0–35.0 g/dl	32.0	31.6	P 3.0–4.5 mg/dL	3.6	4.3
PLT 150–450 K/μL	399	311	Mg 1.6–2.6 mg/dl	1.90	1.97
ESR 0–20 mm	62	47	Bilirubin –total <= 1.4 mg/dl	0.30	0.50
PT%	74	58	Bilirubin-direct 0.00–0.30 mg/dl	0.14	0.21
INR 0.85–1.15 INR	1.18	1.35	SGOT < 40 U/l	8	15
APTT 25–35 sec	21.9		SGPT 0–41 U/l	15	27
Fibrinogen 200–450 mg/dl	606	488	γ-GT 8–61 U/l	41	25
D-dimers < 0.4 mcg/ml	0.51	0.11	ALP 30–120 units /l	103	98
PLT 150–450 K/μL	399	311	LDH 135–225 U/l	155	170
Antithrombin-3 80–120 %	83		Amylase 40–140 U/l	44	35
Protein C 70–140 %	111		CPK 0–190 U/l	26	146
Free PS 60–140 %	103		Cholesterol < 200 mg/dl	211	
APC Resistance > 2.5 seconds	179.4		LDL < 130 mg/dl	148	
La Ratio < 1.2	1.19		HDL > 65 mg/dl	28	
RPR	Negative		Total Protein 6.4–8.3 gr/dl	7.4	8
HBsAg	Negative		Albumin 3.5–5.2 gr/dl	3.3	3.8
HIV Ag-Ab	Negative		Fe 33–193 μg/dl	22	54
Procalcitonin	0.05		Ferritin 30–400 ng/ml	71	473
CRP < 0.5 mg/dl	5	1.8	TSH 0.27–4.20 μIU/ml	0.44	
ACE 13.3–63.9 U/L	4.7		FT4 12.0–22.0 pmol/l	0.97	
C3 79–152 mg/dl	205		IGG 751–1560 mg/dl	2153	
C4 16–38 mg/dl	40		IGA 82–453 mg/dl	381	
RA test 0–20 IU/ml	5		IGM 46–304 mg/dl	132	
B12 145–569 pmol/l	131		IGE 22–165 IU/ml	168	
Folic acid 8.83–60.8 nmol/l	2.90		C.E.A. < 3.8 ng/ml	2.63	
Anti-ENA screen < 20 RU/ml	Negative		CA 15.3 < 25 U/ml	31.7	
Anti-Cardiolipin IGG	< 2 negative		CA 19.9 < 27 U/ml	20.83	
Anti-Cardiolipin IGM	< 2 negative		CA 125 < 35 U/ml	57.6	
ANA < 1:160	Negative		a-FP < 7.0 ng/ml	1.5	
Anti-ds DNA < 1:10	Negative		Widal	negative	
ANCA-c	< 2 negative		Wright	negative	
ANCA-p	< 2 negative		Uric acid 3.4–7.0 mg/dl	6.3	5.9
β2GPI (ΙgG) < 20 RU/ml	< 2 negative				
β2 GPI (IgΜ) < 20 RU/ml	< 2 negative				
Urine examination					
Color	Yellow		Microscopic examination		
Appearance	Clear		RBC	30–50/HPF	
Specific Gravity	1020		WBC	8–10/HPF	
PH	7				
HGB	25				
Protein	Trace				
Glucose	Normal				
Urobilinogen	Negative				
Nitrogen	Negative				

*WBC*: white blood cell; *RBC*: red blood cells; *PLT*: platelets; *HGB*: hemoglobin; *HCT*: hematocrit; *MCV*: Mean corpuscular volume; *MCH*: mean cell hemoglobin; *MCHC*: mean corpuscular hemoglobin concentration; *ESR*: Erythrocyte Sedimentation Rate; *PT*: Prothrombin Time; *APTT*: Activated partial thromboplastin time; *INR*: international normalized ratio; *SGOT*: Serum Glutamil Oxaloacetic Transaminase; *SGPT*: Serum Glutamic Pyruvic Transaminase; *γGT*: Gamma-Glutamyl Transpeptidase; *LDH*: Lactate Dehydrogenase; *ALP*: alkaline phosphatase; *CPK*: Creatine phosphokinase; *LDL*: low-density lipoprotein; *HDL*: high-density lipoprotein; *free PS*: free protein S; *APC-resistance*: Resistance to activated protein C; *La-ratio*: lupus anticoagulant-ratio; *RPR*: Rapid Plasma Reagin; *CRP*: C-reactive protein; *ACE*: Angiotensin-converting enzyme; *TSH*: thyroid-stimulating hormone; *FT4*: free thyroxine; *RA-test*: rheumatoid arthritis test; *IGG*: Immunoglobulin G; *IGM*: Immunoglobulin M; *IGE*: Immunoglobulin E; *IGA*: Immunoglobulin A; *C3*: Complement Component 3; *C4*: Complement Component 4; *ENA screen*: Extractable Nuclear Antigen Antibodies screen; *CEA*: carcinoembryonic antigen; *CA 15.3*: Cancer Antigen 15.3; *CA 19.9*: Cancer Antigen 19.9; *CA 125*: Cancer Antigen 125; *ANA*: Antinuclear antibody; *a-FP*: a-Fetoprotein; *Anti-ds-DNA*: anti-double stranded DNA; *ANCA*: antineutrophil cytoplasmic antibodies; *β2GPI*: Beta 2 Glycoprotein I

A hematologist evaluated the slight elevation in the ratio of globulins to albumin, which was attributed to infection.

An abdominal CT scan did not reveal any pathological locus. A skin biopsy excluded any other systematic diseases responsible for skin lesions associated with stroke. The histopathological results reported were indicative of Sweet syndrome.

After admission, the patient had dark-colored vaginal discharge and itching in the external genital organs. After 14 days of antibiotic therapy and multiple negative blood and urine cultures for common bacteria, she continued having dark-colored vaginal discharge. All blood and urine cultures were collected at bedside under sterile conditions. Blood cultures were collected each time from two to three different areas, always under fever-free conditions, because the patient never had fever. Cultures were performed for common aerobic and anaerobic bacteria, and fungi.

Further serum antibody tests were ordered for Listeria monocytogenes, Borrelia burgdorferi, Mycoplasma pneumoniae (MP) and Ureaplasma ureolyticum. No evidence of infection was found. The Mantoux skin reaction was also negative.

A culture of the vaginal discharge obtained during obstetric examination tested positive for *Mycoplasma hominis*. Antibiotic therapy was prescribed with doxycycline, isoconazole nitrate and diflucortolone valerate for external use, as well as fluconazole because of *Candida non-albicans *co-infection.

The patient was also examined for Fabry disease (a-galactosidase, lyso-GL-3) through tandem mass spectrometry from a dried blood spot, which was also negative.

After recovery, the patient gradually became mobile, and a psychological change was apparent, including emotional instability, fabricated memories, sexual delirium and a lack of inhibition.

Further tests for autoimmune encephalitis with serum antibodies and paraneoplastic antibodies yielded negative results.

The patient was discharged in a fully mobile state but remained incapable of vertical eye movements. She had blepharoptosis and esophoria of the right eye and persistent mild diplopia. Her main issue was psychiatric symptoms, such as disinhibition, mainly affecting speech with logorrhea, inappropriate jokes and emotional instability, which persisted in discharge and remained present at the 3-month follow-up. Her discharge medication included doxycycline at 200 mg/day twice orally for 14 days; isoconazole nitrate and diflucortolone valerate for external use for 7 days; and fluconazole at 150 mg/day orally for 14 days because of Candida non-albicans co-infection. During her hospitalization, she also received oral metoprolol at 100 mg, ¼ b.i.d. because of her persisting tachycardia after cardiology consultation, acetylsalicylic acid at 100 mg once daily for secondary stroke prevention, folic acid at 5 mg once daily, hydroxocobalamin IM once/month, omeprazole at 40 mg once daily, risperidone at 1 mg ½ t.i.d., sertraline at 100 mg once daily, given for her neuropsychiatric symptoms. She was advised to continue these medications at discharge. We were informed that her follow up visit to her personal obstetrician-gynecologist after the end of the antibiotic treatment yielded normal findings, and the repeated cultures were negative.

In the recent 2-year follow-up, the patient's behavior had improved, no other cerebrovascular events had occurred, and her medication was modified to rosuvastatin at 10 mg once daily, acetylsalicylic acid at 100 mg once daily, lisinopril at 20 mg once daily, lansoprazole at 20 mg once daily and quetiapine at 25 mg once daily.

## Discussion

Our patient experienced a stroke with an unspecified cause despite our many laboratory and imaging tests. She had a concurrent genital infection with *Mycoplasma hominis* for an unknown duration. The role of *Mycoplasma hominis* in stroke development in the presence of urogenital tract infections remains unknown. This mollicute has a complex relationship with the host immune response [3]. *Mycoplasma hominis* has not been reported to be associated with stroke in the existing literature. However, multiple literature descriptions exist of strokes post-*Mycoplasma pneumoniae* (MP) infection, particularly in the pediatric population [4–6]. Because both bacteria belong to the same family, they might share a similar pathogenic correlation with stroke. In previously described MP infections, the possible explanations for this correlation include local vasculitis mediated by cytokines and chemokines (IL-6, IL-8, IL-18 and TNF-a), thrombotic vascular occlusion and hypercoagulability induced by MP, which is associated with surface proteins and chemical mediators produced by MP [5, 6] in the absence of a systematic hypercoagulable state.

The progression of cerebral infarction to the right cerebellar hemisphere despite antiplatelet treatment and close monitoring observed in our patient has also been described in a case report of a 5-year-old boy with MP infection [4]. The patient’s behavioral changes were attributed to the thalamic infarcts, as previously described [7, 8]. The disinhibition, mainly affecting speech with logorrhea, inappropriate jokes and emotional instability, persisted after discharge and remained present at the 3-month follow-up.

Smoking is a common risk factor for stroke. However, smoking rarely causes stroke at such young age. A literature meta-analysis suggested that smoking patients with ischemic stroke are 10 years younger than nonsmoking patients at the time of the first onset of stroke [9]; however, none of the studies included in the meta-analysis described patients under 40 years of age. Moreover, recent data suggest that quitting smoking has a positive effect on the incidence of stroke [10]. Our patient was a heavy smoker, and we believe that smoking was a detrimental neuroinflammatory factor contributing to the occurrence of stroke.

The association between infection and stroke is bidirectional. Although infection can lead to stroke, stroke also induces immune suppression, which in turn increases the risk of infection and may result in poorer overall post stroke outcomes. Increasing evidence indicates that the aggregate burden of chronic and/or past infections rather than any single infectious disease is associated with the risk of stroke [11]. Increased systemic inflammation may in itself increase stroke risk and magnify the effect of conventional stroke risk factors [12]. Our patient had a genital infection of unknown origin; we believe that this chronic infection, which persisted for at least 1 month during hospitalization, was associated with an inflammatory response that co-determined the risk of stroke. Genitourinary infections have been reported as risk factors in peripartum stroke, yet they may be an underrecognized factor precipitating peripartum stroke [13]. Notably, infection may play a role in triggering postpartum ischemic stroke even in the absence of other risk factors [14].

## Conclusion

The management of mycoplasma-associated stroke remains controversial [5]. *Mycoplasma hominis* infection and a causal correlation with stroke are difficult to determine, because no previous cases have been described in the literature. However, infection has been described as a well-supported risk factor for stroke, and several potential pathophysiological mechanisms have been suggested. Infections in patients with stroke should be treated fast and prevented in the post-stroke period, because they may result in poorer overall post stroke outcomes.

## Data Availability

Data sharing does not apply to this article, because no datasets were generated or analyzed during the current study.
